# *N*-(2-hydroxypropyl)methacrylamide–amphotericin B (HPMA–AmB) copolymer conjugates as antileishmanial agents

**DOI:** 10.1016/j.ijantimicag.2008.10.013

**Published:** 2009-05

**Authors:** Salvatore Nicoletti, Karin Seifert, Ian H. Gilbert

**Affiliations:** a Division of Biological Chemistry and Drug Discovery, College of Life Sciences, University of Dundee, Sir James Black Centre, Dundee DD1 5EH, UK; b London School of Hygiene and Tropical Medicine, Keppel Street, London WC1E 7HT, UK

**Keywords:** Leishmaniasis, Copolymers, Amphotericin B

## Abstract

Leishmaniasis is a major health problem in many parts of the world, caused by various species of *Leishmania*. Amastigotes are the clinically relevant form of the parasite in the human host and reside in the parasitophorous vacuole within macrophages. Polymer–drug conjugates have been used for lysosomotropic drug delivery and have already shown potential in anticancer and antileishmanial chemotherapy. We synthesised *N*-(2-hydroxypropyl)methacrylamide–amphotericin B (HPMA–AmB) copolymer conjugates in which the AmB was attached to the polymer through a degradable GlyPheLeuGly linker. Antileishmanial activity was assessed in vitro against intracellular amastigotes in host macrophages [murine peritoneal exudate macrophages (PEMs), murine bone marrow-derived macrophages (BMMs) and differentiated THP-1 cells]. The most potent copolymers had 50% effective concentration (EC_50_) values of 0.03 μg/mL AmB equivalent against *Leishmania donovani* amastigotes in PEMs and BMMs and an EC_50_ of 0.57 μg/mL AmB equivalent against *L. donovani* in THP-1 cells. This activity was comparable with free AmB (EC_50_ = 0.03–0.07 μg/mL against *L. donovani* in PEMs and BMMs and 0.24–0.42 μg/mL against amastigotes in THP-1 cells) and Fungizone^®^ (EC_50_ = 0.04–0.07 μg/mL against amastigotes in PEMs). Conjugates also showed potent in vivo activity with ca. 50% inhibition of parasite burden at 1 mg/kg body weight.

## 1 Introduction

Leishmaniasis is a disease complex caused by obligate intracellular protozoa of the genus *Leishmania.* The most serious form of disease is the systemic infection visceral leishmaniasis (VL) (kala-azar), which is fatal unless treated. Other disease manifestations are cutaneous leishmaniasis and mucocutaneous leishmaniasis, which are severely debilitating and disfiguring. There are an estimated 500 000 new VL cases each year, with >90% of cases occurring in India, Bangladesh, Nepal, Sudan, Brazil and Ethiopia [1,2]. Current first-line treatment for VL is focused on pentavalent antimonials (Pentostam^®^ and Glucantime^®^), or conventional amphotericin B (amphotericin B deoxycholate; Fungizone^®^) in areas with high antimony treatment failure rates. Liposomal amphotericin B, miltefosine and paromomycin have become available in recent years as alternative treatments [2].

Amphotericin B (AmB) is a polyene antibiotic, originally developed as a systemic antifungal, that is highly active against *Leishmania* spp. However, major clinical drawbacks of treatment are its toxic side effects, the need for extensive monitoring and a complicated dosing regimen [2–4]. Lipid formulations of AmB have been developed to improve tolerability and efficacy and have been successfully applied in VL [3]. The liposomal formulation AmBisome^®^ has been shown to cure 90% of VL patients in India with single-dose therapies of 5 mg/kg and 7.5 mg/kg [5,6]. However, high cost has limited the wider use of AmBisome^®^, especially in low-income countries with the highest burden of disease, although the price has recently been reduced for the public health sector in VL endemic countries [2,5,7].

The *Leishmania* parasite has a dimorphic life-cycle and within the host resides as the amastigote stage in tissue macrophages within a vacuole, called the parasitophorous vacuole (PV). The PV has many similarities to late endosomes/lysosomes, for example low pH (ca. 5), many of the degradative enzymes associated with lysosomes such as cathepsins B, D, H and L, as well as other proteins typical of lysosomes and late endosomes such as lamp1 and rab7p [8–10]. Thus, drug delivery systems for leishmaniasis that employ active and passive macrophage targeting strategies have been investigated [11]. Polymer–drug conjugates have been used for lysosomotropic drug delivery in the field of anticancer therapy [12–15]. Cell entry is restricted to endocytosis, and polymer conjugates are subsequently trafficked through endosomes to lysosomes [16]. By attaching a drug to the polymer via lysosomally degradable linkers, such as the GlyPheLeuGly (GFLG) linker, drugs can be specifically released in lysosomes [12,17].

*N*-(2-hydroxypropyl)methacrylamide (HPMA) copolymers have shown promise in antileishmanial drug delivery of an 8-aminoquinoline [18,19]. HPMA is water soluble and non-immunogenic and has been in clinical trials in the cancer field [14]. Other polymer–drug conjugates that showed potential in antileishmanial drug delivery include conjugates of antimonials with dextrin [20] and AmB with arabinogalactan [21–23].

We chose to study the delivery of AmB as a model drug based on its known efficacy and toxicity. In this paper, we report the synthesis and biological evaluation of HPMA-GFLG-AmB and HPMA-GFLG-AmB-mannosamine (ManN) conjugates as potential antileishmanial agents.

## 2 Experimental design

### 2.1 Materials, instruments and methods

Polymeric HPMA containing 9.03 mol% GFLG linker activated as the *para-*nitrophenol ester (ONp) (HPMA-GFLG-ONp) was purchased from Polymer Laboratories Ltd. (Church Stretton, UK). Triethylamine was distilled from potassium hydroxide and stored under nitrogen. AmB with a content of amphotericin A <5% was purchased from A.G. Scientific, Inc. (San Diego, CA). Other reagents and solvents were purchased from Aldrich and Fluka. All reactions were carried out at room temperature under an argon atmosphere.

Normal-phase thin-layer chromatography (TLC) was performed on pre-coated sheets of silica 60F_254_, using methanol as the mobile phase (*R*_f_ of AmB = 0.31; *R*_f_ of ONp and of the polymer = 0.9). Reverse-phase TLC was carried out using pre-coated aluminium sheets reverse-phase 18F_254s_ from Merck, using a mixture of KH_2_PO_4_ (0.025 M, pH 5.0)–acetonitrile [9:1] as a mobile phase and ninhydrin as the stain (*R*_f_ of ManN = 0.72; *R*_f_ of ONp and of the polymer = 0.9). Sephadex™ LH-20 was purchased from Fisher Scientific.

Proton nuclear magnetic resonance (^1^H-NMR) spectra were recorded on a Bruker Avance DPX 500 spectrometer in CD_3_OD. All the signals are described as broad (br). UV-Vis spectra were recorded on a Beckman DU 640 spectrophotometer. Solid phase extraction (SPE) high-performance liquid chromatography (HPLC) analyses were performed using a Dionex UltiMate 3000 HPLC instrument, using a μBondapack C_18_ 10 μm 125 Å, 300 mm × 4.6 mm I.D. column (Waters). SPE was carried out using Bond Elut^®^ C18 cartridges of 3 mL capacity containing 100 mg of stationary phase (Varian).

### 2.2 Synthesis of HPMA-GFLG-AmB conjugates (e.g. CIR1592)

A solution of AmB [251.4 mg dissolved in 3.0 mL of dimethyl sulphoxide (DMSO), 0.26 mmol] and triethylamine (38.0 μL, 0.27 mmol) were added to a stirred solution of HPMA-GFLG-ONp (277.4 mg, 0.14 mmol ONp) in dry DMSO (1.6 mL), with monitoring by normal-phase TLC. After 5 h, more triethylamine (38.0 μL, 0.27 mmol) was added. The resulting mixture was stirred overnight, quenched with an excess of 1-amino-2-propanol (20.0 μL), precipitated with diethyl ether and centrifuged. The pellet was dissolved in a minimum amount of methanol and purified by gel filtration using LH-20 as stationary phase and methanol as eluent. The product was a yellow foam, 191.0 mg (yield based on polymer weight, 68.8%); *δ*_H_ 1.03–5.99 (br, H of HPMA, glycine, leucine, phenylalanine and AmB); 6.31 (br, H of double bonds in AmB); 7.31 and 7.54 (br, H of Ph in phenylalanine).

### 2.3 Synthesis of HPMA-GFLG-ManN-AmB conjugate (CIR1669)

d-mannosamine hydrochloride (68.2 mg dissolved in 5.0 mL of DMSO) and triethylamine (43.0 μL) were stirred with a solution of HPMA-GFLG-ONp (945.41 mg, 0.47 mmol ONp) in dry DMSO (5.0 mL) for 4 h, with monitoring by reverse-phase TLC. Then, AmB (415.0 mg dissolved in 10.0 mL of DMSO, 0.45 mmol) and triethylamine (61 μL, 0.44 mmol) were added. The resulting solution was stirred for 5 h, with monitoring by normal-phase TLC. More triethylamine (61.0 μL, 0.44 mmol) was added, the mixture was stirred overnight and quenched with an excess of 1-amino-2-propanol (70.0 μL). Further steps, as for CIR1592, gave the product CIR1669 as a yellow foam, 601 mg (yield based on polymer weight, 63.6%); *δ*_H_ 1.03–5.99 (br, H of HPMA, glycine, leucine, phenylalanine, AmB and ManN); 6.36 (br, H of double bonds in AmB); 7.32 and 7.53 (br, H of Ph in phenylalanine).

### 2.4 Synthesis of controls

HPMA-GFLG-COOH (CIR1465), HPMA-GFLG-1-amino-2-propanol (HPMA-GFLG-AP) (CIR1466) and HPMA-GFLG-ManN (CIR1770) were prepared similarly by treating HPMA-GFLG-ONp with sodium hydroxide, 1-amino-2-propanol and d-mannosamine hydrochloride, respectively.

### 2.5 Determination of the ManN content in HPMA-GFLG-ManN-AmB by a modified Elson and Morgan method [18,19,24,25]

Samples (0.5 mg) were hydrolysed with 6 N HCl (final volume 1.0 mL) at 60 °C for 5 h and then dried under vacuum. The residue was dissolved in 0.5 mL of water and 0.5 mL of acetyl acetone reagent [4.8% (v/v) acetyl acetone in a buffer of 1.0 M sodium bicarbonate and 1.0 M sodium carbonate, pH 9.6]. The resulting mixtures were stirred for 20 min at 96 °C. Subsequently, the solutions were cooled in ice and 2.5 mL of absolute ethanol and 0.5 mL of Ehrlich's reagent (0.8 mg of *p*-dimethylaminobenzaldehyde in 30 mL of absolute ethanol and 30 mL of concentrated HCl) were added and stirred for 10 min at 65 °C. Absorbance was measured at *λ* = 530 nm. A calibration curve was obtained using mixtures of d-mannosamine hydrochloride (17.6–58.7 μg/mL) and HPMA-GFLG-AP (0.5 mg/mL).

### 2.6 Determination of the total amphotericin B content in HPMA-GFLG-AmB and in HPMA-GFLG-ManN-AmB by UV-Vis spectroscopy

A calibration curve was prepared using AmB in DMSO in the range 1.1–10.3 μg/mL by recording the absorbance at 416 nm (maximum absorbance). This was then used to calculate the total amount of AmB associated with the polymer conjugates. HPMA copolymer control (CIR1465) in the same concentration range as the analysed conjugate was used as blank.

### 2.7 Determination of the free amphotericin B content in HPMA-GFLG-AmB and in HPMA-GFLG-ManN-AmB by SPE-HPLC using 1-amino-4-nitronaphthalene as internal standard

C18 cartridges attached to a Vac-Elut chamber were conditioned by passing 3 mL of MeOH and 2 mL of water later at a flow rate of 1 mL/min. Methanolic sample solutions were loaded to the SPE columns at the flow rate of 0.2 mL/min and washed with 1 mL of water at a flow rate of 1 mL/min to elute the copolymer. The retained free residual AmB and internal standard (IS) were eluted with 4 mL of MeOH. The methanolic solutions were dried under vacuum. The obtained residues were dissolved in 1.0 mL of a mixture of ethylene diamine tetra-acetic acid (EDTA) (20.0 mM solution in water) and acetonitrile (CH_3_CN) (60:40 v/v).

HPLC conditions were: eluent EDTA (20.0 mM in water) and acetonitrile (60:40 v/v); a flow rate of 1.0 mL/min; retention time for AmB of 10.7 min; retention time for the IS of 16.9 min; injection volume 100 μL; and detection at 405 nm (maximum absorbance). The calibration curve was obtained using mixtures of AmB (0.5–2.6 μg/mL), HPMA-GFLG-AP (0.2 mg/mL) and IS (10 μg/mL) by least-squares linear regression analysis. The peak area ratio of AmB to IS versus nominal concentration of the drug was plotted. No impurities were detected during these analyses.

### 2.8 In vitro antileishmanial activity against intracellular amastigotes

*Leishmania donovani* (MHOM/ET/67/L82) was maintained in Syrian hamsters and amastigotes were harvested from the spleen of an infected animal. Murine peritoneal exudate macrophages (PEMs) were prepared as described previously [26]. THP-1 cells were differentiated by incubation in medium containing 20 ng/mL phorbol 12-myristate 13-acetate (PMA) (Sigma) for 2 days [27] and rested in PMA-free medium overnight before infection. Bone marrow-derived macrophages (BMMs) were harvested from BALB/C mice according to a standard protocol with minor modifications [28]. Cells were incubated in Dulbecco's Modified Eagle's Medium (DMEM) (Sigma) supplemented with 10% heat-inactivated fetal calf serum (hi-FCS) and 15% L929 cell supernatant at 37 °C in 5% CO_2_ in humidified air for 8 days. Macrophages were harvested and plated as above.

Adherent PEMs, BMMs and THP-1 cells were infected with *L. donovani* amastigotes as described previously [26]. Stock solutions of free AmB and HPMA-GFLG-AmB conjugates were prepared in 100% DMSO (Sigma) at 1 mg/mL AmB equivalent. Fungizone^®^ and AmBisome^®^ were reconstituted according to the manufacturer's protocol at stock concentrations of 5 mg/mL and 4 mg/mL AmB. Subsequent dilutions were prepared in RPMI 1640 medium plus 10% hi-FCS. Maximum DMSO concentrations of 0.1% (PEMs and BMMs) and 0.5% (THP-1 cells) had no effect on parasite clearance. Two hundred microlitres of three-fold serially diluted drug and conjugate solutions were added to the respective wells and each concentration was tested in quadruplicate.

Infected cultures were incubated for 72 h at 37 °C in a 5% CO_2_–95% humidified air mixture. At the experimental endpoint, slides were fixed with 100% methanol and stained with 10% Giemsa in water. Drug and conjugate activity was determined from the percentage of infected macrophages in relation to a non-treated control upon microscopic counting of 100 macrophages per well. Data were analysed by non-linear sigmoidal curve fitting, and 50% effective concentration/90% effective concentration (EC_50_/EC_90_) values were estimated using Microsoft XLfit (ID Business Solution, Guildford, UK). Two to three separate experiments were performed.

### 2.9 In vitro cytotoxicity testing

KB cells (HeLa contaminant, cervical adenocarcinoma-derived) were plated in 96-well plates (Becton Dickinson) at a density of 4 × 10^4^ cells/mL in RPMI 1640 medium + 10% hi-FCS. Cells were allowed to adhere overnight at 37 °C in 5% CO_2_ in humidified air. For THP-1 cells, serial dilutions of drugs and conjugates were prepared in 96-well plates and a suspension of THP-1 cells was added at a seeding density of 5 × 10^5^ cells/mL.

Stock solutions of HPMA-GFLG-AmB conjugates were prepared at 20 mg/mL AmB equivalent in 100% DMSO and subsequent three-fold serial dilutions in culture medium. Concentrations tested ranged from 200 μg/mL to 0.82 μg/mL AmB equivalent (maximum DMSO concentration 1%) for KB cells and from 90 μg/mL to 0.37 μg/mL AmB equivalent (maximum DMSO concentration 0.45%) for THP-1 cells. Each concentration was tested in triplicate. Cultures were incubated for 72 h at 37 °C in a 5% CO_2_–95% air mixture. Twenty microlitres of Alamar Blue^®^ were added for the last 4 h (KB) or 6 h (THP-1) of incubation and plates were read on a Gemini Spectrophotometer (Molecular Devices Ltd.), with excitation at 530 nm and emission at 580 nm.

Data were graphically expressed as percentage cell viability of control calculated as follows: (FI test agent dilution/FI untreated control) × 100, where FI is the fluorescence intensity emission unit.

### 2.10 Statistical analysis

Statistical analysis was performed employing an unpaired, two-tailed *t*-test assuming equal variance. A *P*-value of <0.05 was considered statistically significant.

### 2.11 In vivo antileishmanial activity

Female BALB/c mice were infected by injection of 2 × 10^7^ amastigotes/0.2 mL of medium into the tail vein and were randomly sorted. Solutions of HPMA-GFLG-AmB conjugates were prepared in phosphate-buffered saline (Sigma). AmBisome^®^ (Gilead Sciences) was reconstituted according to the manufacturer's protocol and subsequent dilutions were prepared in 5% dextrose. Groups of five mice were dosed intravenously with doses indicated below on Days 7, 9 and 11 post infection with a bolus injection of 0.2 mL using a 25 gauge needle. Mice were monitored for any overt signs of toxicity (e.g. hunched backs, piloerection, inactive behaviour) and group weights were recorded before and after treatment as a gross indicator of toxicity. On Day 14 post infection, mice were euthanised, their livers were weighed and impression smears were taken. Smears were fixed in 100% methanol, stained in 10% Giemsa in water and the number of amastigotes per 500 host cell nuclei was counted. Leishman–Donovan units (LDU) were calculated using the formula: LDU = number of parasites per host cell nucleus × organ weight in mg [29]. Deviations from the number of host cell nuclei given above were taken into account and corrected for by changing the reference base.

The reduction in parasite burden achieved in a particular animal was calculated relative to the mean LDU (*n* = 5) of the control group and expressed as percentage inhibition. In dose–response experiments, 50% and 90% effective dose (ED_50_ and ED_90_) values were estimated using Microsoft XLfit as described above.

## 3 Results

### 3.1 Preparation of the polymer–drug conjugates

**Fig. 1:**
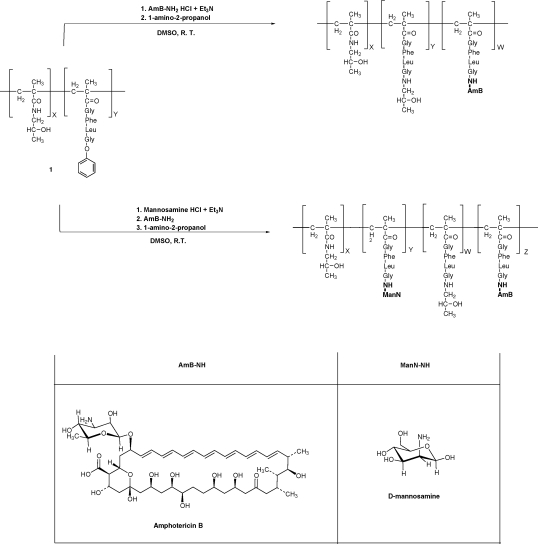
Schematic representation of the synthesis of HPMA-GFLG-AmB and HPMA-GFLG-ManN-AmB. HPMA, *N*-(2-hydroxypropyl)methacrylamide; GFLG, GlyPheLeuGly linker; AmB, amphotericin B; ManN, mannosamine.

A library of HPMA-GFLG copolymer conjugates was prepared containing AmB with or without mannose as a targeting moiety (Fig. 1; Table 1). The GFLG linker was chosen as it is known to be cleaved by cathepsin B [12,16,17], which has been shown to be found in the PV [8]. The prepared conjugates were purified by gel filtration (using LH-20) to remove unreacted AmB. The free residual unbound AmB of the purified copolymers was <1% w/w of the total AmB (Table 1). Quantification of mannose loading was carried out using a modified Elson and Morgan method [24,25].

### 3.2 Evaluation of antileishmanial activity of polymer–drug conjugates against intracellular *Leishmania donovani* amastigotes and cytotoxicity against mammalian cells

HPMA-GFLG-AmB conjugates were evaluated with respect to: (i) activities against intracellular amastigotes in different host macrophages; (ii) influence of drug loading on antileishmanial activity; (iii) influence of mannosylation on antileishmanial activity and (iv) cytotoxicity against two mammalian cell lines.

Conjugates were biologically evaluated against intracellular *L. donovani* amastigotes in PEMs, BMMs and differentiated THP-1 cells. Conjugates displayed activity of the same order of magnitude as AmB and Fungizone^®^ against intracellular *Leishmania* amastigotes (Table 2). *Leishmania* amastigotes residing in differentiated THP-1 cells appeared less susceptible to AmB than amastigotes in primary macrophages (PEMs and BMMs), whether it was used as the free compound, AmB deoxycholate (Fungizone^®^) or conjugated to polymers. At the EC_50_ level, this difference was 4–8-fold for free AmB and Fungizone^®^. A higher difference was observed for HPMA-GFLG-AmB conjugates and this attained factors of 9–67-fold in repeated experiments.

To assess the influence of drug loading, CIR1592 (27.5 wt% AmB) was compared with CIR1668 (9.6 wt% AmB). CIR1592 was more potent against intracellular amastigotes in PEMs than the conjugate CIR1668, and this was statistically significant in repeated experiments (*P* < 0.05). However, there was no significant difference between CIR1592 and CIR1668 against amastigotes in BMMs. No clear picture emerged for amastigotes in differentiated THP-1 cells, where in one experiment CIR1668 was more active than CIR1592 (*P* < 0.05) and in a second experiment CIR1592 showed higher activity than CIR1668 (data not shown).

To investigate the impact of mannosylation on antileishmanial activity, we compared CIR1668 (9.6 wt% AmB, no mannosylation) and CIR1669 (10.4 wt% AmB, 5.9 wt% ManN) in all three *Leishmania*–host cell models. Both conjugates displayed similar activity, with no advantage of mannosylation observed (Table 2).

**Fig. 2:**
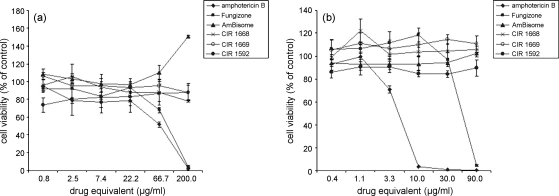
Cytotoxicity of amphotericin B (AmB), Fungizone^®^ and HPMA–AmB conjugates (CIR1668, CIR1669 and CIR1592) against (a) KB cells (b) and monocytic THP-1 cells in suspension. Data are presented as % cell viability of control. Drug equivalent refers to AmB equivalent. Data represent the arithmetic mean ± standard error of the mean (*n* = 3). HPMA, *N*-(2-hydroxypropyl)methacrylamide.

All conjugates displayed a favourable cytotoxicity profile compared with the free drug and Fungizone^®^, and comparable with AmBisome^®^. No toxicity was observed against KB cells up to 200 μg/mL AmB equivalent (Fig. 2a) or against monocytic THP-1 cells in suspension up to 90 μg/mL AmB equivalent (Fig. 2b). In contrast, Fungizone^®^ and AmB started to show toxicity against KB cells between 22.2 μg/mL and 66.7 μg/mL, AmB against THP-1 cells between 1.1 μg/mL and 3.3 μg/mL and Fungizone^®^ against THP-1 cells at ca. 90 μg/mL.

### 3.3 Evaluation of HPMA-GFLG-AmB and HPMA-GFLG-ManN-AmB in a mouse model of infection

Based on demonstrated antileishmanial activity and lack of cytotoxicity in vitro, in vivo studies were conducted in BALB/c mice. Initially, mice were dosed with conjugate CIR1592 (27.5% AmB) at 3 mg/kg and 1 mg/kg body weight intravenously (i.v.) × 3 and AmBisome^®^ was included as comparator. CIR1592 showed excellent activity at both doses and caused inhibition of parasite burden of 99.6% at 3 mg/kg i.v. × 3 and 93.8% at 1 mg/kg i.v. × 3. For comparison, AmBisome^®^ caused an inhibition of 99.9% at both doses (experiment 1, Table 3).

Based on this result, a dose–response experiment was performed comparing CIR1592 with AmBisome^®^ at doses of 1, 0.3 and 0.1 mg/kg i.v. × 3. Although AmBisome^®^ remained more active, with an ED_50_ of 0.2 mg/kg body weight, the experiment confirmed high in vivo activity of CIR1592 with a predictive ED_50_ value of ca. 1 mg/kg body weight (experiment 2, Table 3). There is a slight difference in the efficacy of CIR1592 between experiment 1 and experiment 2; however, it should be noted that in experiment 1 levels of parasitaemia were approximately two-fold lower than in experiment 2.

To investigate the effect of the mannose targeting moiety on parasite reduction in vivo, the antileishmanial activities of CIR1668 and CIR1669 were compared in the BALB/c mouse model. CIR1668 and CIR1669 conjugates suppressed hepatic parasite burden by 97.9% and 99.3% at 3 mg/kg i.v. × 3, respectively, and by 46.6% and 34.6% at 1 mg/kg i.v. × 3. No significant difference in antileishmanial activity was found between the conjugate with and without mannose (experiment 3, Table 3). Importantly, no signs of toxicity in vivo were recorded during administration of the HPMA-GFLG-AmB conjugates at doses given, which is comparable with AmBisome^®^ (Table 3).

## 4 Discussion

There is an urgent need to improve therapy for leishmaniasis. Exploring drug delivery systems is one way to circumvent problems of drug toxicity and potentially to increase antileishmanial drug efficacy. We assessed the potential of HPMA–AmB drug conjugates as antileishmanial agents in vitro and in vivo. AmB was chosen as it is an important drug for the treatment of leishmaniasis but suffers limitations. Conventional AmB in the form of a deoxycholate salt (Fungizone^®^) displays adverse reactions and requires administration under medical surveillance. A liposomal formulation of AmB (AmBisome^®^) has an improved safety profile but its use has been limited by cost. HPMA has shown potential in antileishmanial drug delivery [18,19] and much information is available in the anticancer field [12,14].

On a cellular level, HPMA-GFLG-AmB conjugates showed potent activity against intracellular *L. donovani* amastigotes in a panel of macrophages in vitro and displayed decreased cytotoxicity against two mammalian cell lines. There was some variability in activity between different batches of polymer conjugates, but there was significant activity against intracellular *Leishmania* in all batches tested. We chose to study three different types of macrophages based on previous observations that different AmB formulations displayed different activities in the PEM and THP-1–amastigote models [30] as well as early studies that demonstrated differences in macrophage populations support of parasite growth. BMMs were found to be more permissive than resident peritoneal macrophages [31] and represent natural host cells of *Leishmania*. *Leishmania* amastigotes in different types of macrophages showed different susceptibilities to AmB, whether given as the free drug, a liposomal formulation or as the polymer conjugate. This difference was particularly noticeable between primary macrophages (PEMs and BMMs) and a monocytic cell line (THP-1 cells) and might be explained by differences in uptake and internalisation. Internalisation of AmB has been shown to occur via endocytosis and also through uptake of AmB–low-density lipoprotein (LDL) complexes via the LDL receptor pathway [32–34]. Given the endocytic route of internalisation described for polymeric carriers, differences in endocytic/phagocytic potential of primary macrophages and macrophage-like THP-1 cells could be underlying reasons for this observation.

Mannosylation has been used as an active targeting principle in macrophage-specific drug delivery and experimental antileishmanial chemotherapy [11,18,19,35,36], which prompted us to investigate mannose as a targeting moiety on the HPMA-GFLG-AmB conjugates. However, we found no advantage of mannose loadings at ca. 6%. The lack of a significant difference in activity between the non-mannosylated and mannosylated conjugate may be due to conformational hindrance that prevents interaction of the polymer-bound mannose with the mannose receptor. Alternatively, the rate of uptake through other mechanism(s) may be significantly faster than that through mannose receptor-mediated endocytosis.

The most important findings are that (i) HPMA-GFLG-AmB conjugates showed potent antileishmanial activity in vivo, although less active than AmBisome^®^ but of a similar order to it and (ii) that no signs of toxicity were recorded, even at the maximum dose of 3 mg/kg AmB equivalent used. It should be noted that this dose is above the reported 50% lethal dose (LD_50_) of 2.5 mg/kg for Fungizone^®^ [37]. This clearly confirms the improved toxicity profile of conjugates observed in vitro and demonstrates the same effect in vivo. It means that conjugates are less toxic but the reported ED_50_ is between 0.5 mg/kg and 1 mg/kg so the efficacy is comparable. The predictive ED_50_ for CIR1592 was five-fold higher than the ED_50_ of AmBisome^®^. However, it is known that AmBisome^®^ is taken up by macrophages of the reticuloendothelial system and preferentially distributed to the liver and spleen, which are the target organs of VL, and that therapeutic doses remain for several weeks after loading doses [38].

Further work is required to optimise these HPMA-GFLG-AmB copolymer conjugates, including the investigation of mode of action, different linkers, biodistribution and pharmacokinetic data. However, the results presented here are a promising proof of principle for further studies in this area.

*Funding*: This research was funded by the Wellcome Trust (grant 074136).

*Competing interests*: None declared.

*Ethical approval*: All animal experiments were conducted under licence in accordance with UK Home Office approval.

## Figures and Tables

**Table 1:** Polymers prepared and evaluated.

Name	Conjugate	Total ManN (% w/w)^a^	Total AmB (% w/w)^b^	Free AmB (% total drug)^c^
	HPMA-GFLG-ONp	–	–	–
CIR1592	HPMA-GFLG-AmB	–	27.5	<0.5
CIR1668	HPMA-GFLG-AmB	–	9.6	<0.1
CIR1669	HPMA-GFLG-ManN-AmB	5.9	10.4	<1.0
CIR1770	HPMA-GFLG-ManN	5.9	–	–
CIR1465	HPMA-GFLG-COOH	–	–	–
CIR1466	HPMA-GFLG-AP			

ManN, mannosamine; AmB, amphotericin B; HPMA, *N*-(2-hydroxypropyl)methacrylamide; GFLG, GlyPheLeuGly linker; ONp, *para-*nitrophenol ester; AP, 1-amino-2-propanol.

a The content of ManN in the conjugates CIR1669 and CIR1770 was determined by a modified Morgan and Elson method.

b The content of total Am B in the conjugates (CIR1592, CIR1668 and CIR1669) was determined by UV-Vis analysis.

c Free residual AmB, reported as weight % of the total loaded drug, was determined by high-performance liquid chromatography (HPLC) analysis.

**Table 2:** In vitro activity of amphotericin B (AmB), Fungizone^®^ and HPMA–AmB conjugates against intracellular *Leishmania donovani* amastigotes in different host macrophages.

Polymer/drug	PEMs			BMMs			THP-1 cells		
	EC_50_	EC_90_	% inf.	EC_50_	EC_90_	% inf.	EC_50_	EC_90_	% inf.
Fungizone^®^	0.04 (0.03–0.05)	0.10 (0.09–0.11)	92 ± 2	n.o.	0.02 (0.02–0.03)	72 ± 8	n.o.	0.22 (0.20–0.24)	95 ± 1
AmB	0.06 (0.05–0.07)	0.17 (0.12–0.23)	92 ± 2	0.03 (0.02–0.04)	0.05 (0.03–0.06)	72 ± 8	0.24 (0.22–0.26)	0.48 (0.45–0.52)	95 ± 1
CIR1592 (HPMA-GFLG-AmB)	0.03 (0.02–0.03)	0.09 (0.07–0.10)	92 ± 2	0.03 (0.02–0.04)	0.06 (0.05–0.07)	72 ± 8	1.09 (0.92–1.26)	4.01 (1.83–6.20)	95 ± 1
CIR1668 (HPMA-GFLG- AmB)	0.07 (0.05–0.09)	0.71 (0.21–1.21)	92 ± 2	0.03 (0.02–0.04)	0.08 (0.05–0.11)	72 ± 8	0.63 (0.58–0.68)	2.50 (1.26–3.73)	95 ± 1
CIR1669 (HPMA-GFLG- ManN-AmB)	0.05 (0.02–0.07)	0.33 (0.17–0.49)	92 ± 2	0.03 (0.03–0.03)	0.15 (0.02–0.28)	72 ± 8	0.57 (0.51–0.62)	1.99 (1.60–2.38)	95 ± 1

HPMA, *N*-(2-hydroxypropyl)methacrylamide; PEMs, peritoneal exudate macrophages; BMMs, bone marrow-derived macrophages; EC_50/90_, 50% and 90% effective concentration, respectively; % inf., % of infected macrophages at the end of the experiment in untreated controls (± standard deviation); n.o., not obtained. Polymers without AmB (CIR1465, CIR1466 and CIR1770) were included at the top polymer concentration used and showed no significant inhibition. Data given as EC_50_/EC_90_ values in μg/mL AmB equivalent (with 95% confidence intervals in parentheses).

**Table 3:** Activity of HPMA-GFLG-AmB conjugates in comparison with AmBisome^®^ in BALB/c mice infected with *Leishmania donovani*.

Polymer/drug	Dose (mg/kg) × 3^a^	% inhibition^b^	ED_50_ (mg/kg)	ED_90_ (mg/kg)	LDU^b^
Experiment 1					
CIR1592	3	99.6 ± 0.1	n.d.	n.d.	590 ± 72
	1	93.8 ± 1.6	n.d.	n.d.	590 ± 72

AmBisome^®^	3	99.9 ± 0.1	n.d.	n.d.	590 ± 72
	1	99.9 ± 0.1	n.d.	n.d.	590 ± 72

Experiment 2					
CIR1592	1	51.9 ± 7.7	0.97	>1	1105 ± 141
	0.3	13.0 ± 5.8			1105 ± 141
	0.1	9.8 ± 9.3			1105 ± 141

AmBisome^®^	1	98.7 ± 0.7	0.22	0.77	1105 ± 141
	0.3	60.9 ± 5.9			1105 ± 141
	0.1	22.8 ± 5.9			1105 ± 141

Experiment 3					
CIR1668	3	97.9 ± 1.0	n.d.	n.d.	1249 ± 194
	1	46.6 ± 6.1	n.d.	n.d.	1249 ± 194

CIR1669	3	99.3 ± 0.2	n.d.	n.d.	1249 ± 194
	1	34.6 ± 9.4	n.d.	n.d.	1249 ± 194

HPMA, *N*-(2-hydroxypropyl)methacrylamide; GFLG, GlyPheLeuGly linker; AmB, amphotericin B; ED_50/90_, 50% and 90% effective dose, respectively; LDU, Leishman–Donovan units at experimental endpoint. HPMA copolymers were included as control at the top dose used. Parasite inhibition caused by CIR1465 and CIR1466 in experiment 1 was 42.2 ± 5.4% and 35.1 ± 7.7% at a dose of 3 mg/kg body weight × 3. No inhibition was observed at a dose of 1 mg/kg body weight × 3 in experiment 2.

a Doses are given in mg/kg body weight and were administered on Days 7, 9 and 11 post infection.

b % inhibition and LDUs are given as arithmetic mean ± standard error of the mean (*n* = 5).

## References

[1] Guerin P.J., Olliaro P., Sundar S., Boelaert M., Croft S.L., Desjeux P. (2002). Visceral leishmaniasis: current status of control, diagnosis, and treatment, and a proposed research and development agenda. Lancet Infect Dis.

[2] Chappuis F., Sundar S., Hailu A., Ghalib H., Rijal S., Peeling R.W. (2007). Visceral leishmaniasis: what are the needs for diagnosis, treatment and control?. Nat Rev Microbiol.

[3] Sundar S., Mehta H., Suresh A.V., Singh S.P., Rai M., Murray H.W. (2004). Amphotericin B treatment for Indian visceral leishmaniasis: conventional versus lipid formulations. Clin Infect Dis.

[4] Mueller Y., Nguimfack A., Cavailler P., Couffignal S., Rwakimari J.B., Loutan L. (2008). Safety and effectiveness of amphotericin B deoxycholate for the treatment of visceral leishmaniasis in Uganda. Ann Trop Med Parasitol.

[5] Sundar S., Jha T.K., Thakur C.P., Mishra M., Singh V.P., Buffels R. (2003). Single-dose liposomal amphotericin B in the treatment of visceral leishmaniasis in India: a multicenter study. Clin Infect Dis.

[6] Sundar S., Agrawal G., Rai M., Makharia M.K., Murray H.W. (2001). Treatment of Indian visceral leishmaniasis with single or daily infusions of low dose liposomal amphotericin B: randomised trial. BMJ.

[7] Bern C., Adler-Moore J., Berenguer J., Boelaert M., den Boer M., Davidson R.N. (2006). Liposomal amphotericin B for the treatment of visceral leishmaniasis. Clin Infect Dis.

[8] Lang T., Hellio R., Kaye P.M., Antoine J.C. (1994). *Leishmania donovani*-infected macrophages—characterization of the parasitophorous vacuole and potential role of this organelle in antigen presentation. J Cell Sci.

[9] Russell D.G., Xu S.M., Chakraborty P. (1992). Intracellular trafficking and the parasitophorous vacuole of *Leishmania mexicana*-infected macrophages. J Cell Sci.

[10] Courret N., Frehel C., Gouhier N., Pouchelet M., Pina E., Roux P. (2002). Biogenesis of *Leishmania*-harbouring parasitophorous vacuoles following phagocytosis of the metacyclic promastigote or amastigote stages of the parasites. J Cell Sci.

[11] Basu M.K., Lala S. (2004). Macrophage specific drug delivery in experimental leishmaniasis. Curr Mol Med.

[12] Duncan R. (2003). The dawning era of polymer therapeutics. Nat Rev Drug Discov.

[13] Duncan R., Budman D., Calvert H., Rowinsky E. (2003). Polymer–drug conjugates. Handbook of anticancer drug development.

[14] Vincent M.J., Duncan R. (2006). Polymer conjugates: nanosized medicines for treating cancer. Trends Biotechnol.

[15] Brocchini S., Duncan R., Mathiowitz E. (1999). Pendent drugs, release from polymers. Encyclopaedia of controlled drug delivery.

[16] Vincent M.J., Greco F., Nicholson R.I., Paul A., Griffiths P.C., Duncan R. (2005). Polymer therapeutics designed for a combination therapy of hormone-dependent cancer. Angew Chem Int Ed Engl.

[17] Duncan R., Cable H.C., Lloyd J.B., Rejmanova P., Kopacek J. (1984). Polymers containing enzymatically degradable bonds, 7. Design of oligopeptide side chains in poly [*N*-(2-hydroxylpropyl)methacrylamide] copolymers to promote efficient degradation by lysosomal enzymes. Makromol Chem.

[18] Nan A., Croft S.L., Yardley V., Ghandehari H. (2004). Targetable water-soluble polymer–drug conjugates for the treatment of visceral leishmaniasis. J Control Release.

[19] Nan A., Dhammika N.P., Walker L.A., Yardley V., Croft S.L., Ghandehari H. (2001). *N*-(2-hydroxypropyl)methacrylamide (HPMA) copolymers for targeted delivery of 8-aminoquinoline antileishmanial drugs. J Control Release.

[20] Demicheli C., Ochoa R., da Silva J.B.B., Falcao C.A.B., Rossi-Bergmann B., de Melo A.L. (2004). Oral delivery of meglumine antimoniate–β-cyclodextrin complex for treatment of leishmaniasis. Antimicrob Agents Chemother.

[21] Golenser J., Frankenburg S., Ehrenfreund T., Domb A.J. (1999). Efficacious treatment of experimental leishmaniasis with amphotericin B–arabinogalactan water-soluble derivatives. Antimicrob Agents Chemother.

[22] Sokolsky-Papkov M., Domb A.J., Golenser J. (2006). Impact of aldehyde content on amphotericin B–dextran imine conjugate toxicity. Biomacromolecules.

[23] Ehrenfreund-Kleinman T., Golenser J., Domb A.J. (2004). Conjugation of amino-containing drugs to polysaccharides by tosylation: amphotericin B–arabinogalactan conjugates. Biomaterials.

[24] Configliacchi E., Razzano G., Rizzo V., Vigevani A. (1996). HPLC methods for the determination of bound and free doxorubicin, and of bound and free galactosamine, in methacrylamide polymer–drug conjugates. J Pharm Biomed Anal.

[25] Jang J.H., Hia H.C., Ike M., Inoue C., Fujita M., Yoshida T. (2005). Acid hydrolysis and quantitative determination of total hexosamines of an exopolysaccharide produced by *Citrobacter* sp.. Biotechnol Lett.

[26] Seifert K., Croft S.L. (2006). In vitro and in vivo interactions between miltefosine and other antileishmanial drugs. Antimicrob Agents Chemother.

[27] Tsuchiya S., Kobayashi Y., Goto Y., Okumura H., Nakae S., Konno T. (1982). Induction of maturation in cultured human monocytic leukemia cells by a phorbol diester. Cancer Res.

[28] Coligan J.E., Kruisbeek A.M., Margulies D.H., Shevach E.M., Strober W. (2003). Current protocols in immunology.

[29] Bradley D.J., Kirkley J. (1977). Regulation of *Leishmania* populations within the host. I. The variable course of *Leishmania donovani* infections in mice. Clin Exp Immunol.

[30] Yardley V., Croft S.L. (2000). A comparison of the activities of three amphotericin B lipid formulations against experimental visceral and cutaneous leishmaniasis. Int J Antimicrob Agents.

[31] Crocker P.R., Davies E.V., Blackwell J.M. (1987). Variable expression of the murine natural resistance gene *Lsh* in different macrophage populations infected in vitro with *Leishmania donovani*. Parasite Immunol.

[32] Brajtburg J., Bolard J. (1996). Carrier effects on biological activity of amphotericin B. Clin Microbiol Rev.

[33] Legrand P., Vertut-Doi A., Bolard J. (1996). Comparative internalization and recycling of different amphotericin B formulations by a macrophage-like cell line. J Antimicrob Chemother.

[34] Vertut-Doï A., Ohnishi S.I., Bolard J. (1994). The endocytic process in CHO cells, a toxic pathway of the polyene antibiotic amphotericin B. Antimicrob Agents Chemother.

[35] Roberts W.L., Hariprashad J., Rainey P.M., Murray H.W. (1996). Pentavalent antimony–mannan conjugate therapy of experimental visceral leishmaniasis. Am J Trop Med Hyg.

[36] Negre E., Chance M.L., Hanboula S.Y., Monsigny M., Roche A.C., Mayer R.M. (1992). Antileishmanial drug targeting through glycosylated polymers specifically internalized by macrophage membrane lectins. Antimicrob Agents Chemother.

[37] Larabi M., Yardley V., Loiseau P.M., Appel M., Legrand P., Gulik A. (2003). Toxicity and antileishmanial activity of a new stable lipid suspension of amphotericin B. Antimicrob Agents Chemother.

[38] Adler-Moore J., Proffitt R.T. (2003). Effect of tissue penetration on AmBisome efficacy. Curr Opin Investig Drugs.

